# The Role of Cofactors in Prion Propagation and Infectivity

**DOI:** 10.1371/journal.ppat.1002589

**Published:** 2012-04-12

**Authors:** Jiyan Ma

**Affiliations:** Department of Molecular and Cellular Biochemistry, Ohio State University, Columbus, Ohio, United States of America; Washington University School of Medicine, United States of America

## Introduction

The term “prion" was originally coined by Prusiner to explain the unusual infectious agent in transmissible spongiform encephalopathies (TSEs, also known as prion disease) [1]. Now the term has expanded to include a growing list of fungal proteins that stably maintain an atypical self-propagating conformation and epigenetically modify a variety of cellular processes [2]. Although fungal prions and the TSE agent share the capability of maintaining an atypical self-propagating conformation, fungal prions distinctly differ from the TSE agent in several aspects [3]. Thus far, the TSE agent is the only prion that behaves as a bona fide infectious agent, having an infectious cycle, capable of transmitting horizontally (among a community) and causing epidemic outbreaks [3]. The discussion in this article will be focused on mammalian prion, and the term “prion" specifically refers to the infectious TSE agent.

## Prion Is a Protein Conformation-Based Infectious Agent

Prions were defined as small proteinaceous infectious particles that cause TSEs [1], a group of fatal neurodegenerative diseases including Creutzfeldt-Jakob disease (CJD) in human, scrapie in sheep, bovine spongiform encephalopathy (BSE) in cattle, and chronic wasting disease (CWD) in deer and elk. Prion protein (PrP) is an N-linked glycoprotein tethered to lipid membranes via a glycophosphatidylinositol (GPI)-anchor, widely expressed in various tissues and highly enriched in the central nervous system. The host-encoded normal prion protein (PrP^C^) is α-helical rich, soluble in mild detergents, sensitive to protease digestion, and releasable from lipid membrane by phosphatidylinositol-specific phospholipase C (PI-PLC) digestion. During prion disease, a portion of PrP converts to an aberrant conformational isoform called PrP^Sc^, which is mostly β-sheeted, highly aggregated, protease-resistant, and unable to be released from lipid membrane by PI-PLC digestion.

The prion hypothesis postulates that, because of its self-propagating property, PrP^Sc^ isoform seeds the conversion of normal PrP^C^ to the pathogenic PrP^Sc^ and causes the disease. The self-propagating characteristic of disease-associated PrP^Sc^ has been demonstrated by cell-free conversion assay and serial protein misfolding cyclic amplification (sPMCA) [4], [5]. The simultaneous propagation of protease-resistant PrP^Sc^ and prion infectivity in sPMCA provides strong evidence supporting the prion hypothesis [5]. Because crude brain homogenate is used in sPMCA for prion propagation, it is difficult to conclusively establish a causal relationship between PrP^Sc^ and prion disease.

Clearly establishing that PrP^Sc^, an altered conformational PrP isoform, is the causative agent for prion disease has been a challenge for the prion field for decades, mainly because PrP^Sc^ is highly aggregated, preventing it from being purified to homogeneity using conventional biochemical methods. An alternative approach is to reconstitute prion infectivity in vitro with defined components. Amyloid fibers formed in vitro with bacterially expressed recombinant PrP (recPrP) have been shown to contain limited prion infectivity [6]–[8]. Infectious prions have been formed through unseeded sPMCA with native PrP^C^ purified from brains [9] or recPrP [10] as substrate, or by converting recPrP into an infectious conformer in sPMCA seeded with partially purified PrP^Sc^ [11]. More importantly, the infectivity of prions generated via sPMCA has been demonstrated by causing bona fide prion disease in wild-type animals [9], [10], [11]. Because bacterially expressed recPrP does not contain any eukaryotic genetic informational molecules, generating prion infectivity with recPrP [10]–[12] is generally accepted as the most stringent proof of prion hypothesis.

## Cofactors Promote Prion Propagation

A key concept of the prion hypothesis is that prion is a self-propagating PrP conformer, which elicits the conversion of host-encoded normal PrP^C^ to pathogenic PrP^Sc^. This self-propagating property of PrP conformers is best demonstrated by in vitro assays. Recombinant PrP in amyloid fibers represents a self-propagating conformational state that is distinct from normal PrP^C^, but recPrP in amyloid fibers is generally without the biochemical hallmark of PrP^Sc^, C-terminal proteinase K (PK)-resistance. The in vitro assays that propagate the classic PK-resistant PrP^Sc^ conformation include cell-free conversion assay and sPMCA [4], [5]. The cell-free conversion uses partially purified PrP^Sc^ as seed and PrP^C^ purified from cultured cells as substrate, which is far less efficient than sPMCA that employs crude brain homogenates. The difference between two assays suggests the presence of factors in the brain homogenates that promote efficient prion propagation. Polyanions, such as RNA molecules and proteoglycans, have been identified as one type of cofactors in the brain homogenate that enhance prion propagation [13], [14]. Lipids are another type of cofactors that promote prion propagation in cell-free conversion assay [15] and in propagating recombinant prions via sPMCA [10].

## Self-Propagating PrP Conformation Does Not Always Correlate to Prion Infectivity

Prion infectivity obviously depends on the seeding or self-propagating property of PrP conformers. However, a PrP conformer with seeding or self-propagating capability does not necessarily contain prion infectivity in vivo, that is, cause spongiform encephalopathy in animals. This notion is exemplified by PrP amyloid fiber, which clearly contains a strong seeding or self-propagating capability, but does not have a strong association with in vivo infectivity. The PrP amyloid fibers in the brain tissues of a familial prion disease patient induce a prominent PrP-amyloid accumulation in “humanized" knock-in mice carrying the same mutation, demonstrating in vivo seeding or self-propagating property of PrP amyloid fibers [16]. However, a lack of spongiform encephalopathy or any neurological disorders during the life span of these PrP amyloid–bearing mice suggests a dissociation of prion infectivity from seeding capability [16]. Similarly, in vitro generated recPrP amyloid fibers contain a strong in vitro seeding capability, but very low prion infectivity [6]–[8]. Even with PK-resistant PrP conformers, their abilities to propagate the PK-resistant conformation in sPMCA do not always associate with in vivo prion infectivity [17].

Collectively, the seeding or self-propagating property of a prion is essential for its infectivity, but not all PrP conformers with seeding or self-propagating capability are competent to cause prion disease in animals.

## Cofactors Appear to Play an Important Role in Prion Infectivity

The role of cofactors in prion infectivity remains to be clarified, but comparing several in vitro reconstituted infectious prions indicates that they play an important role in prion infectivity. When infectious prions were formed with recPrP or purified PrP^C^ via sPMCA in the presence of lipid and RNA as cofactors, the newly formed prions contain high infectivity [9], [10]. When recombinant prions were formed via seeded sPMCA in the absence of any mammalian cofactors, the prion infectivity is lower but still sufficient to cause disease in wild-type animals [11]. Notably, the buffer used in cofactor-free sPMCA contains SDS and Triton X-100 detergents, which are similar to lipid in biophysical properties and may partially replace the function of a lipid cofactor. The in vitro recPrP amyloid fiber formation, however, is in a partially denaturing buffer and completely without any cofactors. Interestingly, recPrP amyloid fibers contain very low infectivity, fail to cause disease in wild-type animals, and only cause disease with prolonged incubation times in PrP overexpressing mice [6], [7]. The limited infectivity of recPrP amyloid fibers in animals could be explained by binding to cofactors in vivo, leading to further PrP adaptations and conformational changes [18]. Nevertheless, the comparison of in vitro reconstituted prions indicates that the presence of cofactor enhances in vivo prion infectivity.

## Mechanism of Cofactor in Prion Propagation and Infectivity

To date, two types of cofactors, lipids and polyanions, are identified as influencing prion propagation and infectivity, but the precise mechanism remains unclear. It can be envisioned that cofactors may act on several steps of PrP conversion. Both polyanion and lipid bindings alter the normal PrP^C^ conformation to increase β-sheet content and PrP aggregation, a conformational change similar to PrP^C^-to-PrP^Sc^ conversion [19]–[21]. Thus, cofactor binding may render the normal PrP^C^ susceptible to conversion or simply facilitate PrP^Sc^-steered PrP^C^ conversion. Alternatively, cofactors may facilitate PrP conversion by concentrating both PrP^Sc^ and PrP^C^ on the surface of a single lipid vesicle or a single polyanion molecule. Another possibility could be that the cofactors stabilize the infectious PrP^Sc^ conformation by forming a complex with PrP^Sc^ and being a part of the infectious particle.

Both lipids and polyanions enhance prion propagation, yet polyanion does not appear to be critical for prion infectivity [22]. Lipids, on the other hand, not only influence prion propagation, but also have a significant impact on prion infectivity. A ∼100-fold increase in prion infectivity has been observed when purified prion is re-incorporated into liposomes [23]. Therefore, these two types of cofactors may act on the same or different steps in prion propagation and infectivity.

Most cofactor-related studies use mouse or hamster prions. It is well known that prions from different species or different prion strains have very different properties, which could be due to the different combinations of various lipid or polyanion molecules. It is also possible that other types of molecules could serve as cofactors, contributing to the diverse biological properties of prions. Further investigation of prion cofactors will help us to gain the insights of this enigmatic infectious agent, which is essential for us to combat these devastating diseases.
